# Comparison of the therapeutic effect between the simultaneous and staged unicompartmental knee arthroplasty (UKA) for bilateral knee medial compartment arthritis

**DOI:** 10.1186/s12891-019-2724-8

**Published:** 2019-07-27

**Authors:** Shuo Feng, Zhi Yang, Jian-Ning Sun, Liang Zhu, Song Wang, Kai-Jin Guo, Xiang-Yang Chen, Guo-Chun Zha

**Affiliations:** Department of Orthopedic Surgery, Affiliated Hospital of Xuzhou Medical University, 99 Huaihai Road, Xuzhou, Jiangsu 221002 People’s Republic of China

**Keywords:** Unicompartmental knee arthroplasty, Simultaneous, Staged, osteoarthritis, Postoperative complications

## Abstract

**Background:**

To compare the efficacy and safety of simultaneous vs staged unicompartmental knee arthroplasty (UKA) for bilateral unicompartmental osteoarthritis of the knee.

**Methods:**

We performed a retrospective analysis of prospectively collected data on 93 patients with bilateral knee medial compartment arthritis underwent simultaneous UKA (Group A, 39) or staged UKA (Group B, 54 cases) from January 2008 to December 2015. Group A: 6 males and 33 females aged 64.9 ± 7.7 years; Group B: 5 males and 49 females aged 64.2 ± 6.4 years. There were no statistically significant differences in pre-operative age, sex ratio, or body weight index between the groups (*P* > 0.05). Groups were compared in terms of total anesthesia time, volume of drainage, blood transfusion rate, hemoglobin level on post-operative day 3, total post-operative inpatient days, treatment expenses, post-operative therapeutic effect (KSS scores), and complications.

**Results:**

All patients had follow-up visits post-operatively. The follow-up visit interval was 32–133 months and 41.9 months on average. Total anesthesia time, postoperative length of hospital stay, and hospitalization expenses in Group A were significantly less than those of Group B (*P* < 0.05). Hemoglobin levels in Group A were significantly lower than those of Group B at post-op day 3 (*P* < 0.05). However, no significant differences in volume of drainage, the rate of transfusion, complications, and KSS scores were detected between Groups A and B (*P* > 0.05).

**Conclusions:**

Both simultaneous and staged UKA achieved the desired therapeutic effect in treatment of bilateral knee medial compartment arthritis. However, simultaneous UKA reduced the cost and the postoperative length of hospital stay without increasing post-operative complications.

## Background

Unicompartmental knee arthroplasty (UKA) can effectively relieve knee pain and improve patients’ quality of life. Compared with total knee replacement (TKA), UKA is less invasive, with less trauma and less bleeding. What’s more, it also shortens the length of hospital stay, and rehabilitation time [1–3]. .Previous studies have shown that 1/3 of patients who underwent TKA suffered from bilateral knee joint degeneration. Twenty percent underwent contralateral TKA within 2 years following unilateral TKA [4–6]. Patients with bilateral arthritis requiring surgical treatment have two options: a staged procedure with a period of time between operations, and a simultaneous procedure, either sequentially by a single surgical team, or simultaneously by two surgical teams. Compared with staged surgery, simultaneous surgery may reduce hospitalization expenses, length of hospital stay, and rehabilitation time [7–10]. In spite of various advantages of simultaneous surgery, some investigators believe that it increases bleeding, operation time, perioperative period complications, revision rate, death rate, and blood transfusion rate [11, 12].

Currently, there are a large number of reports on simultaneous TKA, but few reports on the selection of simultaneous vs staged UKA for bilateral knee joint unicompartmental osteoarthritis [7, 11]. Thus, the preference of simultaneous or staged bilateral UKA and perioperative risks remains unclear. The current study retrospectively analyzes clinical data from UKA patients with bilateral knee unicompartmental osteoarthritis, in order to assess the therapeutic effect and safety of simultaneous vs staged bilateral UKA.

## Materials and methods

### Patient source

The study was a retrospective analysis of prospectively collected data study, approved by institutional review board. Informed consent was obtained from all patients included in the study. All data (surgical records, hospital records) were obtained from hospital computer data, collected by independent personnel. Three patients rejected the second UKA with an exit ratio of 3.1%. A total of 93 patients with bilateral knee unicompartmental osteoarthritis underwent UKA in our department from January 2008 to December 2015. All patients received a 6-month conservative treatment before considering surgery. We divided the patients into two groups according to surgical methods: Group A (simultaneous UKA; patients who underwent bilateral UKA under the same anesthesia, conducted consecutively by one team, 39 cases) and Group B (staged UKA; separate hospitalizations, bilateral UKA, 54 cases).

### General data

See Table 1 for the pre-operative data regarding the two groups of patients.

**Table 1:** Demographic characteristics of patients used in this study

Item	Group A (*n* = 39)	Group B (*n* = 54)	t/χ^2^	*P* Value
Age (years)	64.9 ± 7.7	64.2 ± 6.4	0.492	0.624
Gender (male/female)	6:33	5:49	0.333	0.564
Weight (kg)	62.8 ± 4.3	61.9 ± 4.8	0.955	0.342
Height (cm)	162.6 ± 6.4	162.5 ± 6.5	0.057	0.955
BMI (kg/m^2^)	23.9 ± 2.5	23.5 ± 2.6	0.583	0.561
ASA class	2.1 ± 0.5	2.1 ± 0.5	0.012	0.990
Comorbidity(n, %)	14 (35.9%)	26 (48.1%)	1.387	0.239
Hypertension	7	11		
CAOD	3	4		
Arrhythmia	0	1		
Pulmonary disease	1	2		
Diabetes mellitus	3	7		
CKD	0	0		
CVA	0	1		

*Abbreviations*: *BMI* body mass index, *ASA* American Society of Anesthesiologists, *CAOD* coronary artery obstructive disease, *CKD* chronic kidney disease, *CVA* cerebral vascular accident, *KS* knee society score

### Surgical method

All surgeries were performed by two highly-qualified surgeons in our orthopedics department with 10 years of experience, and assisted by the anesthesiologist with same professional title. All patients underwent surgery under general anesthesia with pneumatic tourniquet. Each patient lay supine with the affected hip flexion angle ranging from 20° to 30°. Affected knee flexion angle was about 120°. The patient was positioned on a special supporting structure. A towel was applied after routine sterilization. The medial parapatellar approach was used. Proximal tibial and distal femoral osteotomies were performed with the Oxford UKA technique. When the surgery proceeded to the femoral side, the femur used an intramedullary positioning rod. After the opening of the medullary cavity, we suck out the tissue in the medullary cavity, and the positioning rod was thinner and the pressure was small when inserted. For this reason, the fat/marrow embolic phenomenon was theoretically reduced. The Oxford UKR prosthesis was installed and fixed with cement in all cases. A drainage tube was placed in the articular cavity. The incision was sutured layer by layer and dressed with elastic bandage.

Group A: Simultaneous procedures were performed by the same surgeon under one anesthetic. When one side was completed, operation on the other side would proceed.

Group B: The patient underwent contralateral surgery on a voluntary basis at 6 weeks - 6 months following unilateral UKA. The mean interval between staged surgeries was 3 months (SD 0.8). The main reason for the interval was patient choice.

### Post-operative treatment

All patients were cared for by same group of internists and all performed guided rehabilitation exercises. A previously published multimodal rapid recovery protocol was followed for all patients. The drainage tube was removed at 2 days following surgery, and post-operative drainage volume was recorded. Active or passive functional training was performed at 1 day following surgery. Low molecular heparin (4100 IU once per day) was injected subcutaneously to prevent deep venous thrombosis. Patients were allowed to bear loads appropriately and were guided through weight-bearing walking exercise with the help of a walking aid at 3 days post-surgery. The patient went home directly after discharged, and we informed the patient about the rehabilitation method. The complications in the perioperative period were recorded within 3 months of the operation. The KSS scores for left and right knees were recorded separately. KSS scores were recorded prior to operation and within 1 year following [13]. Packed red cells were administered to if the patients developed pallor, palpitations and shortness of breath, or if hemoglobin (Hgb) < 7 g/dL.

### Evaluation index

We reviewed operative reports to collect operative times, blood loss, and intraoperative complications. The postoperative length of stay and perioperative complications and hospitalization expenses were obtained by analysis of hospital records. Outcomes during follow-up were evaluated and recorded by independent observers who were blinded to surgical method. The general data included age, gender, height, weight, BMI, ASA score. The follow-up time was 1 month, 3 months, 6 months, 1 year after surgery, and was followed up once a year thereafter. Intra-operative data included total operation time, postoperative length of hospital stay, blood transfusion rate, and hospitalization expenses(All expenses during hospitalization). For the staged UKA group, total operating time was adding the times of the two surgeries. Total postoperative length of hospital stay was the sum of the two postoperative hospitalizations. Total hospitalization expenses are the sum of two hospitalization expenses. We compared the difference in KSS between the final score at 1year post-surgery and the pre-operative score.

### Statistical method

GraphPad Prism 6.0 and SPSS 16.0 statistical software (Statistical Package for the Social Sciences, Chicago, IL, USA) were used for data analysis. Outcomes of the simultaneous and staged interventions were compared. Measurement data were expressed as mean ± standard deviation (SD), groups comparison using t-test. Student’s t test was used to compare quantitative variables as mean age, BMI, total operation time, Knee Society Scores and postoperative length of hospital stay between A group and B group. The different groups were compared regarding the categorical variables by use of the chi-square test or Fisher exact test. The value of α was 0.05. The differences were statistically significant when *P* < 0.05.

## Result

Groups A and B were similar in terms of demographics and comorbidities. There were no statistically significant differences in pre-operative general data. (*P* > 0.05, Table 1).

There were no significant differences in post-operative total drainage volume (*P* > 0.05, Table 2). Hemoglobin levels on post-operative day three were in Group A were significantly lower than those of Group B (*P* < 0.05, Table 2). However, there were no statistically significant differences in the rate of transfusion between Groups A B (*P* > 0.05, Table 2). Total anesthesia time in Group A (120.2 ± 9.7 min) was significantly shorter than that of Group B (141.6 ± 8.7 min) (*P* < 0.05, Table 2). In Group A, the average length of hospital stay was 4.2 ± 0.7 days. Hospitalization expenses were 11294.2 ± 202.4 USD. In Group B, average length of hospital stay was 7.5 ± 1.5 days. Total hospitalization expenses were 12846.0 ± 405.8 USD. The length of hospital stay and hospitalization expenses in Group A were significantly less than those of Group B (*P* < 0.05, Table 2). Compared with stage UKA, simultaneous UKA cut the length of stay in hospital by almost 50%. The simultaneous procedure was 12.1% less costly compared to the staged procedure.

**Table 2:** Clinical results of all patients undergoing medial UKAs in the two groups

Item	Group A (*n* = 39)	Group B (*n* = 54)	t/χ^2^	*P* Value
Amount of drainage (mL)	490.3 ± 101.2	460.9 ± 124.8	1.211	0.229
Haemoglobin (g/L)				
Pre-operation	135.3 ± 8.5	132.3 ± 11.8	1.373	0.173
3-days after operation	106.9 ± 13.8	126.2 ± 8.4	7.734	*P* < 0.01
Blood transfusion (n, %)	1 (2.6%)	0	Fisher	0.419*
Length of stay (days)	4.2 ± 0.7	7.5 ± 1.5	14.192	*P* < 0.01
Hospital costs (RMB)	75849.8 ± 1358.9	86271.2 ± 2725.3	24.236	*P* < 0.01
Duration of anesthesia (min)	120.2 ± 9.7	141.6 ± 8.7	11.008	*P* < 0.01
Complication (n, %)	4 (10.3%)	5 (9.3%)	0.012	0.913
Cardiac complications	0	0		
Deep infection	0	0		
Confusion	0	0		
Stroke	0	0		
Superficial infection	1	1		
Minor wound dehiscence	2	1		
Skin edge necrosis	0	1		
Respiratory	1	2		
Deep-vein thrombosis	0	0		
Pulmonary embolism	0	0		
Death	0	0		

*Fisher’s exact test

No cases of cardiac events, deep tissue infection, pulmonary embolism, deep-vein thrombosis confusion, stroke, or death were recorded. Minor wound complications such as skin margin necrosis and minor wound dehiscence occurred in four patients. These were managed with oral antibiotics and appropriate wound care. Superficial infections were all successfully treated with oral antibiotics. Patients with respiratory complications were referred to respiratory physician for treatment recommendations. There were a total of seven cases of post-operative complications in Group A (10.3%), and 13 cases in Group B (9.3%) (*P* > 0.05, Table 2).

KSS scores prior to surgery and at 1 year post-surgery were compared. The mean postoperative knee score improved significantly in both groups compared with pre-surgical scores. The total left KSS significantly improved in both groups, from 114.9 ± 5.2 versus 114.7 ± 6.4 points preoperatively (*P* > 0.05) to 169.5 ± 6.6 versus 167.1 ± 6.6 points postoperatively, respectively (*P* > 0.05, Table 3). The total right KSS also significantly improved in both groups from 115.9 ± 6.4 versus 114.5 ± 6.7 points preoperatively (*P* > 0.05) to 170.4 ± 6.6 versus 167.8 ± 8.4 points postoperatively, respectively (P > 0.05, Table 3). There was no significant difference between the groups for any parameter. Functional knee rehabilitation in both groups is displayed in Table 3.

**Table 3:** Postoperative functional results

Variable	Group A (*n* = 39)	Group B (*n* = 54)	t/χ^2^	*P-*Value
Left Knee Society Clinical Scores				
Preoperative	50.0 ± 5.4	50.2 ± 5.4	0.180	0.858
Final Follow-up	87.6 ± 6.2	86.9 ± 5.8	0.538	0.592
Left Knee Society Function Scores				
Preoperative	64.9 ± 4.5	64.6 ± 5.3	0.279	0.781
Final Follow-up	81.6 ± 5.0	80.2 ± 4.2	1.525	0.131
Total Left Knee Society Scores				
Preoperative	114.9 ± 5.2	114.7 ± 6.4	0.147	0.884
Final Follow-up	169.5 ± 6.6	167.1 ± 6.6	1.713	0.090
Right Knee Society Clinical Scores				
Preoperative	50.1 ± 4.6	49.6 ± 5.7	0.421	0.675
Final Follow-up	90.1 ± 4.7	88.6 ± 6.3	1.244	0.217
Right Knee Society Function Scores				
Preoperative	65.8 ± 4.0	64.9 ± 4.9	0.988	0.3236
Final Follow-up	80.3 ± 6.0	79.1 ± 5.4	0.943	0.348
Total Right Knee Society Scores				
Preoperative	115.9 ± 6.4	114.5 ± 6.7	1.020	0.310
Final Follow-up	170.4 ± 6.6	167.8 ± 8.4	1.568	0.120

## Discussion

Simultaneous bilateral TKA is thought to increase perioperative complications and mortality rate, and thus remains controversial [14–16]. Compared with TKA, UKA has a range of advantages, including minimal trauma, low hemorrhage volume, low incidence of complications in the perioperative period, and low mortality [17, 18]. Therefore, in theory, it is appropriate to perform simultaneous bilateral UKA. However, there are few published reports on this subject. Our findings suggest that simultaneous UKA for management of bilateral knee medial compartment arthritis is safe and efficacious, with less total anesthesia time, expense and length of hospital stay, without increasing post-operative complications.

The originator of simultaneous bilateral TKA believes the operation has a wide range of advantages over staged procedures: shorter cumulative anesthesia duration, shorter length of hospital stay, convenience of a single operation, simultaneous rehabilitation training for both knees, high post-operative satisfaction, and lower cost [19–21]. Previous studies of simultaneous bilateral UKA found that patients in the simultaneous operative group had short total length of hospital stay, fewer hospital expenses, and reduced cumulative general anesthesia time [7, 11, 22]. Our study finds similar results. The total anesthesia time (120.2 ± 9.7 min) in Group A was significantly shorter than that of Group B (141.6 ± 8.7 min) (*P* < 0.05). Total anesthesia time for simultaneous UKA surgery is 21 min shorter than that of staged UKA surgery. In the study of Chan et al. [11], the cumulative anesthesia time in the simultaneous UKA group was 114 min, vs 129 min in the staged UKA group. We report coincides similar results. The cause of such differences may be clinically relevant. The study by Chan et al. found that three patients (1.9%) in the simultaneous UKA group suffered cardiac complications. These patients had Class III ASA prior to surgery. Two had a history of heart disease. Chan et al. attribute these complications to the prolonged anesthesia time of simultaneous surgery.

In our study, compared with staged UKA, simultaneous UKA cut the length of stay in hospital by almost 50%. The total length of hospital stay in group A was shorter and the patient was hospitalized by an average of 4.2 ± 0.7 days. However, the total length of hospital stay in group B was 7.5 ± 1.5 days. Simultaneous surgery allows the total length of hospital stay of the patients to decrease by 3 days, thus relieving the pressure imposed on the hospital with respect to the number of hospital beds. The hospitalization expenses of the patients in Group A was 1551.7USD (12.1%) less than Group B. The reason for higher hospitalization costs in staged UKA may be due to anesthesia fees charged twice, and to the cost of two preoperative examinations. Chan et al. [11] and Berend et al. [7] also demonstrated that patients who received simultaneous UKA had shorter cumulative lengths of hospital stay than those who received staged UKA. However, the length of hospital stay in our study is substantially longer than that reported by Chan et al. [11]. In Berend’s study [23], 125 patients received 142 UKAs, with an average hospital stay of 1.3 days.121 patients (97%) were discharged home directly. In 23 (18%) cases, home health physical therapy was used. Physical therapy was performed in 95 patients (76%).Only 4 (3%) patients required skilled care or post-discharge rehabilitation stay. The average hospital stay reported in the Akhtar et al. [22] study was 3.5 days. Our study also took longer than European scholars have reported. This may be due to the different discharge criteria used by different institutions. However, in the United States, UKA is a routine outpatient procedure and most patients do not require hospitalization. Bilateral UKA surgery also can be done in the outpatient, and patients only need to stay for 1–2 days. At present, joint replacement surgery has not been performed in our outpatient, mainly because there are no surgical conditions in the outpatient. In China, patients undergoing joint replacement surgery have longer hospital stays. Previously, They refused to be discharged before the surgical suture was removed (14 days after surgery). We routinely place a drainage tube, because if there is a medical dispute caused by complications such as poor wound healing for not placing the drainage tube, the doctor would ultimately lose the lawsuit. Studies of simultaneous bilateral TKA achieved similar results. It is believed that simultaneous surgery under single anesthesia administration provides various benefits, such as shorter cumulative anesthesia time and lower treatment cost [24].

Morbidity and mortality are crucial to consideration of feasibility of simultaneous bilateral joint replacement. In TKA, simultaneous surgery is thought to increase complications and mortality in the perioperative period. For example, the occurrence frequency of myocardial ischemia increases 4–6 fold, and risk of pulmonary embolism increases by 50–80% [14, 25, 26]. However, some investigators [27, 28] believe that differences in baseline data and surgical proficiency may give rise to disparate results. Naranje [29] reported that a 15-min increase in operative time increased the hazard of revision resulting from infection by only 15.6%. Operative time decreases with increasing experience but appears to plateau at approximately 300 surgeries. Our operations were performed by experienced doctors aiming to minimize the difference in surgical outcomes due to operative time. As TKA fundamentally differs from UKA, TKA data cannot be directly compared with UKA results. Compared with TKA, UKA has a wide range of advantages such as less invasiveness, shorter incision, lower hemorrhage volume, and less bone cut. In our study, hemoglobin levels 3 days post-surgery in Group A were significantly lower than those of Group B (*P* < 0.05). However, there are no statistically significant differences in blood transfusion rate between Groups A B (*P* > 0.05). Four patients (10.3%) suffered post-operative complications in Group A, vs 5 patients (9.3%) in Group B. There were no differences in total rate of post-operative complications between the two groups (*P* > 0.05). Berend et al. [7] retrospectively compared 141 patients (282 knees) and found no significant differences in morbidity and mortality in the perioperative period between simultaneous bilateral UKA and staged bilateral UKA. Akhtar et al. [22] reported on 38 patients (76 knees), with ASA III or below, who underwent simultaneous UKA. No patient died and three patients presented with complications (7.9%). One suffered from tibial plateau fracture and the other two suffered from wound infections. There were no significant differences in complications between bilateral simultaneous replacement and staged replacement. Pandit et al. [30], also reported similar complication rates. These studies return complication results similar to ours.

By contrast, Chan et al. [11] found varying complication rates. Their study included 159 patients (318 knees) who underwent simultaneous bilateral UKA and 80 patients (160 knees) who underwent staged bilateral UKA. The ASA scores of all patients were ≤ 3. The oldest patient was aged 85 years. Of these, 13 patients (8.2%) who underwent bilateral UKA presented with major complications. Nine (5.7%) suffered proximal DVT requiring a long period of anticoagulation. One patient (0.6%) died as a result of a pulmonary embolism. No complications are found in the staged replacement UKA group. Therefore, the Chan group counsels caution regarding simultaneous bilateral UKA. Chan reports that this incidence of pulmonary embolism in simultaneous bilateral UKA is roughly equivalent to the incidence of 0.3–0.8% incidence seen in simultaneous bilateral TKA [14–16]. However, Chan’s group did not employ DVT prophylaxis, which may account for their result. In the case data included in the present study, low-molecular weight heparin or Rivaroxaban was given as DVT prophylaxis. No thromboembolic complications or deaths occurred in our patients.

At one-year follow-up, there were no statistically significant differences in KSS scores between Groups A and B (*P* > 0.05, Table 3). However, Berend et al. [7] found that, at the 90 day follow-up visit, the ratio of the average score of the knee joint functions of the patients who received simultaneous UKA to that of the patients who received staged UKA was 87.9:72.9, *P* < 0.001. The ratio of the lower extremity activity score (LEAS) of the patients who received simultaneous UKA to that of the patients who received staged UKA was 11.3:10.2 (*P* < 0.001). The simultaneous bilateral UKA group was clearly superior to the staged bilateral UKA group. Thus, simultaneous bilateral UKA can shorten overall rehabilitation and early-stage functional results. Powell [31] and Leonard [32] found similar results in the follow-up visits for patients who received simultaneous bilateral total knee replacement. Their patients were highly satisfied with the surgical result.

This study has several limitations: (1) This is a retrospective study, which may have compromised the analysis. Selection bias is the main concern. Secondly, power for occurrence of complications is difficult. We need RCT’s to really answer these questions. (2) despite the data being collected over a 7-year period, there are relatively small sample sizes in each group. While ipsilateral UKAs are less common than TKA, this is probably due to the fact it is much more difficult to find patients who are candidates for bilateral UKAs. (3) simultaneous UKA was performed only in younger patients with good overall health. No clinical investigations, evaluations, or analyses were conducted for older patients.

## Conclusions

In conclusion, both the simultaneous and staged UKA treatment of bilateral knee medial compartment arthritis can achieve desired therapeutic results. Simultaneous UKA decreases the expenses and length of hospital stay without increasing post-operative complications.

## Data Availability

All data generated or analyzed during this study are included in this published article,and the supplementary file. We do not wish to share our patients’ data because it involves patient’s privacy.

## Abbreviations

TKA: Total knee arthroplasty
UKA: Unicompartmental knee arthroplasty
